# Determinants of birth asphyxia among newborns delivered in public hospitals of West Shoa Zone, Central Ethiopia: A case-control study

**DOI:** 10.1371/journal.pone.0248504

**Published:** 2021-03-16

**Authors:** Guta Kune, Habtamu Oljira, Negash Wakgari, Ebisa Zerihun, Mecha Aboma

**Affiliations:** 1 Ambo University Referral Hospital, Ambo, Ethiopia; 2 Department of Public Health, College of Medicine and Health Sciences, Ambo University, Ambo, Ethiopia; 3 Department of Midwifery, College of Medicine and Health Sciences, Ambo University, Ambo, Ethiopia; Population Council, INDIA

## Abstract

Birth asphyxia is one of the leading causes of death in low and middle-income countries and the prominent cause of neonatal mortality in Ethiopia. Early detection and managing its determinants would change the burden of birth asphyxia. Thus, this study identified determinants of birth asphyxia among newborns delivered in public hospitals of West Shoa Zone, central Ethiopia. A hospital-based unmatched case-control study was conducted from May to July 2020. Cases were newborns with APGAR (appearance, pulse, grimaces, activity, and respiration) score of <7 at first and fifth minute of birth and controls were newborns with APGAR score of ≥ 7 at first and fifth minute of birth. All newborns with birth asphyxia during the study period were included in the study while; two comparable controls were selected consecutively after each birth asphyxia case. A pre-tested and structured questionnaire was used to collect maternal socio-demographic and antepartum characteristics. The pre-tested checklist was used to retrieve intrapartum and fetal related factors from both cases and controls. The collected data were entered using Epi-Info and analyzed by SPSS. Bi-variable logistic regression analysis was done to identify the association between each independent variable with the outcome variable. Adjusted odds ratio (AOR) with a 95% CI and a p-value of <0.05 was used to identify determinants of birth asphyxia. In this study, prolonged labor (AOR = 4.15, 95% CI: 1.55, 11.06), breech presentation (AOR = 5.13, 95% CI: 1.99, 13.21), caesarean section delivery (AOR = 3.67, 95% CI: 1.31, 10.23), vaginal assisted delivery (AOR = 5.69, 95% CI: 2.17, 14.91), not use partograph (AOR = 3.36, 95% CI: 1.45, 7.84), and low birth weight (AOR = 3.74, 95% CI:1.49, 9.38) had higher odds of birth asphyxia. Prolonged labor, breech presentation, caesarean and vaginal assisted delivery, fails to use partograph and low birth weights were the determinants of birth asphyxia. Thus, health care providers should follow the progress of labor with partograph to early identify prolonged labor, breech presentation and determine the mode of delivery that would lower the burden of birth asphyxia.

## Introduction

The neonatal period is a very delicate stage of life due to the risk of acquiring potential life-threatening diseases, and the complexity of the adaptive process [1, 2]. World Health Organization has defined birth asphyxia as a failure to start and maintain breathing at birth [3]. Worldwide, an estimated 2.5 million newborns died in the first month of life in 2017 and about 36% died the same day they were born [4].

Globally, intrapartum-asphyxia is responsible for about 814,000 deaths per year, and it is the fifth cause of death in under-five children and also associated with significant morbidity, ensuing in a burden of 42 million disability-adjusted life years [5]. The proportion of birth asphyxia is 2 per 1000 births in developed countries and it is more than 10 times higher in low-income countries with limited access to quality obstetrics care during pregnancy, intrapartum and postpartum period [6]. Studies done in Nigeria [7], Nepal [8], and Bangladesh [9] reported that perinatal asphyxia is the cause of 23.9%, 30%, and 39% of newborns’ deaths respectively.

Ethiopia’s rate of neonatal mortality is still among the highest in sub-Saharan Africa [10]. In Ethiopia, neonatal death decreased by 10% between 2005 and 2016, but to some extent increased to 30 per 1000 live birth in 2019 [11, 12]. The combined prevalence of perinatal asphyxia was 22.8% [13]. Previous studies conducted in Dilla and Tigray, Ethiopia indicated that the prevalence of birth asphyxia was 32.8% and 22.1% respectively [14, 15]. Birth asphyxia is reported as a prominent cause of neonatal death and forms about 31.6%, followed by preterm birth (21.8%), and sepsis (18.5%) [16]. Although the effects of the majority of asphyxia are transient, it has also long-term neurodevelopment sequel, including cognitive and motor disabilities which are almost untreatable [17]. Studies indicated that the survivors of birth asphyxia developed hypoxic-ischemic encephalopathy, neurologic disability, low cognitive function, neurological sequel, hearing, and visual impairment [18–24]. Prenatal obstetric complications, parity, multiple pregnancies, gestational age , birth weight, premature rupture of membranes, prolonged labor, and fetal distress were previously identified as the determinants of birth asphyxia [7–9, 14, 15, 17, 24–30].

Ethiopia has made significant progress in reducing neonatal morbidity and mortality by implementing a comprehensive national child survival strategy in 2015 and guidelines to treat birth asphyxia [11, 30–32]; however, birth asphyxia is still the leading cause of neonatal deaths in the country [33, 34]. Determinants of birth asphyxia might vary across the regions and particularly in a country with varied culture, socio-economic characteristics, and delays in reaching the health facility and receiving care are challenging [33–36]. Ethiopia is a country with poor infrastructure and quality intrapartum care which could influence the quality of obstetrics care and neonatal outcomes [35, 36]. Even though earlier studies identified determinants of birth asphyxia in some parts of Ethiopia [14, 15, 24, 37–39], there is limited data in the study setting taking the differences of socio-economic status, place of residence, regions, culture, and access to obstetrics and neonatal care in the country, which is useful to craft contextual interventions [35, 36]. Hence, this study aimed to identify the determinants of birth asphyxia among newborns delivered in Public Hospitals of West Shoa Zone, Central Ethiopia.

## Materials and methods

### Study design and setting

A hospital-based prospective unmatched case-control study was conducted among newborns delivered from May 20 to July 31, 2020, at Public Hospitals of West Shoa Zone. Ambo is a Zonal town and lies about 114 km from Addis Ababa; the capital city of the country. Based on the current data, the total population of the zone is 2,661,188. It has 22 districts and 572 Kebele of which 53 Urban and 529 Rural. It has 8 public hospitals, 91 health centers, 1 private higher clinic, 40 medium private clinics, 168 small clinics, 43 drug stores, 21 drug vendors, and 4 pharmacies. Eight public hospitals have provided institutional delivery and neonatal intensive care unit services.

### Study participants

All newborns delivered in public hospitals of West Shoa Zone during the data collection period were the source population. In this study, subjects were categorized into cases and controls. Newborn with APGAR scores of <7 at first and fifth minutes after delivery was defined as having birth asphyxia, while newborns with APGAR scores of ≥7 at first and fifth minutes were considered as not having birth asphyxia. Newborns with one or multiple congenital malformations incompatible with life such as hydrops, cyanotic congenital heart defects, and anencephaly were excluded from the study.

### Sample size determination and sampling technique

The sample size was calculated using the Epi-Info for the unmatched case-control study using the following assumptions: 95% confidence level, power 80%, the odds ratio of 2.75, proportion (p) of controls with prolonged labor 31.1% [37], and 2:1 controls to cases ratio. Accordingly, with a 10% non-response rate, the final sample size was 177 (59 cases and 118 controls). From 8 public hospitals in West Shoa Zone, 5 were selected by the lottery method and proportional allocations were done based on the previous year’s two months record of birth asphyxia. All newborns with birth asphyxia during the study period were included in the study while; two comparable controls were selected consecutively after each birth asphyxia case.

### Data collection tools and procedures

A pre-tested and structured interviewer-administered questionnaire was used to collect maternal socio-demography and antepartum characteristics. Intrapartum and fetal characteristics data were extracted by using a pre-tested and structured checklist from the medical records of the women. The questionnaire was adapted from previous studies and contextualized based on its objectives [7–9, 14, 15, 24–29, 37–39]. The questionnaire was prepared in English and translated to the local language, Afan Oromo, and retranslated to English to check for inconsistency. The quality of the questionnaire was assured by properly designing and pre-testing the tool, and training the data collectors and supervisors before the actual data collection. The data collection tool was pre-tested on 9 live births at Ambo General Hospital before the actual data collection period and necessary modifications were made based on the nature of gaps identified in the questionnaire. To verify the internal consistency of the tool, Cronbach’s alpha analysis of antepartum, intrapartum, and fetal related instrument parts was performed with the value of 0.69 which implies that the tool items have internal consistency. Five midwives trained for 2 days to collect the data and two senior midwives participated in the supervision. Birth asphyxia was determined using the components of the APGAR score table. The score comprised five components as appearance (colour), heart rate, grimaces (reflexes), activity (muscle tone), and respiration. For each component, a score of 0, 1, or 2 was given. A score of ≥7 indicated a newborn was not asphyxiated and in a good condition, whereas a low score (<7) indicated newborns have birth asphyxia [40].

### Operational definitions

**Prolonged labor**: When the labor after the latent phase of the first stage of labor exceeds 12 hours in primigravida or 8 hours in multipara mothers [41]. **Premature rupture of the membrane**: This is defined as a condition in which rupture of the membrane of the amniotic sac and chorion occurs 1 hour before the onset of labor [41]. **Anemia**: In pregnant women defined when the hematocrit level is < 33%. **Birth weight**: Was classified as normal when it is between 2,500-4000gm and low if <2500gm [12]. **Mid upper arm circumference** (MUAC): This is used to measure maternal nutritional status and classified as: MUAC≤ 18cm severe malnutrition, 19-22cm moderate malnutrition, and ≥ 23cm normal among pregnant women [42].

### Data analysis

Data were coded and entered into Epi-info version 7 and analyzed by SPSS version 22. Descriptive statistics were computed for independent variables. Multicollinearity among the independent variables was assessed by using variance inflation factors. However, no significant multicollinearity was detected as the variance inflation factor for all variables was less than five. Hosmer and Lemeshow goodness of fit test was used to check model fitness before running the final model. Bi-variable logistic regression analysis was conducted to see the association of each independent variable to the outcome variable and variables with p-values of < 0.25 were identified and fitted to the multivariable logistic regression through the backward stepwise method to reduce the effects of confounders and to identify the independent effects of each variable on the outcome variable. Adjusted odds ratio with 95% CI was computed to identify the presence and strength of associations and statistical significance was declared at a p-value of <0.05.

### Ethics statement

The ethical clearance was obtained from the Ambo University, College of Medicine and Health Sciences research review and ethical committee with the reference number of PGC/54/2020. Then officials at different levels of the selected hospitals had been communicated through a cooperation letter written from the college. The responsible bodies at each delivery ward and mothers of newborns were informed about the purpose of the study. Written informed consent was obtained from mothers of newborns to confirm willingness. They were told that all data used in this study was strictly kept private and confidential. Data collectors were told the mother as they would have the right to participate, refuse and withdraw from the study at any time during the interview, and the failure to participate in the study did not result in a penalty.

## Results

### Socio-demographic characteristics of participants

A total of 174 (58 cases and 116 controls) participated in the study with a response rate of 98.3%. The mean age of the mothers with cases and controls were 26.9 (SD ± 6.4) and 25.9 (SD ± 5.4) years respectively. Out of the total, 41(70.7%) of the mothers with cases and 74(63.8%) of the mothers with controls were rural dwellers. Regarding educational status, 14(24.1%) of the mothers with cases and 36(31.1%) of mothers with controls had no formal education. About 12(20.7%) of the cases and 36(31.1%) of controls had MUAC measurement of 19-22cm (Table 1).

**Table 1:** Socio-demographic characteristics of the mothers of cases and controls, West Shoa Zone, Central Ethiopia, 2020.

Variables	Cases (n = 58)	Controls (n = 116)	Total count (n = 174)
	N (%)	N (%)	N (%)
Maternal age (in years)			
20–34	48(82.7)	100(86.2)	148(85.1)
35–49	10(17.2)	16(13.8)	26(14.9)
Marital status			
Single	4(6.9)	3(2.5)	7(4.0)
Married	50(86.2)	108(93.1)	158(90.8)
Divorced	4(6.9)	5(4.3)	9(5.1)
Ethnicity			
Oromo	44(75.8)	100(86.2)	144(82.7)
Amhara	9(15.5)	15(12.9)	24(13.8)
Gurage	5(8.6)	1(0.8)	6(3.4)
Religion			
Orthodox	24(41.3)	40(34.5)	64(36.7)
Protestant	23(39.6)	52(44.8)	75(43.1)
Muslim	8(13.8)	22(18.9)	30(17.2)
Wakefata	2(3.4)	3(2.5)	5(2.8)
Residence			
Urban	17(29.3)	42(36.2)	59(33.9)
Rural	41(70.7)	74(63.8)	115(66.1)
Educational status			
No formal education	14(24.1)	36(31.0)	50(28.7)
Primary	15(25.8)	27(23.3)	42(24.2
Secondary	16(27.6)	33(28.5)	49(28.1)
College and above	13(22.5)	20(17.2)	33(18.9)
MUAC(in centimeter)			
≤18(severe)	0	8(6.8)	8(4.6)
19-22(moderate)	12(20.7)	28(24.1)	40(23)
≥23(normal)	46(79.3)	80(68.9)	126(72.4)
Height (in centimetre)			
< 153 (short stature)	8(13.8)	16(13.8)	24(13.8)
≥153 (normal)	50(86.2)	100(86.2)	146(86.2)

### Antepartum related characteristics of the study participants

Among study participants, 37(63.8%) of the mothers with cases and 73(62.9%) of the mothers with controls were multiparous. In addition, 7(12.1%) of the mothers with cases and 21(18.1%) of the mothers with controls had no antenatal visit. Four (6.8%) of mothers with cases and 12(10.4%) of mothers with controls had anemia (Table 2).

**Table 2:** Antepartum characteristics of the study participant, West Shoa Zone, Central Ethiopia, 2020.

Variables	Cases (n = 58)	Controls (n = 116)	Total (n = 174)
	N (%)	N (%)	N (%)
Parity			
Primiparous	21(36.2)	43(37.1)	64(36.8)
Multiparous	37(63.8)	73(62.9)	110(63.2)
Previous adverse pregnancy outcome			
Yes	14(24.2)	27(23.3)	41(23.6)
No	44(75.8)	89(76.7)	133(76.4)
Abortion			
Yes	6(10.4)	12(10.4)	18(10.4)
No	52(89.6)	104(89.6)	156(89.6)
Stillbirth			
Yes	2(3.5)	12(10.4)	14(8.1)
No	56(96.5)	104(89.6)	160(91.9)
Child death			
Yes	4(6.9)	7(6.1)	11(6.3)
No	54(93.1)	109(93.9)	163(93.7)
Preterm			
Yes	2(3.5)	5(4.3)	7(4.1)
No	56(96.5)	111(95.7)	167(95.9)
Antenatal care visit			
Yes	51(87.9)	95(81.9)	146(83.9)
No	7(12.1)	21(18.1)	28(16.1)
Number of antenatal care visit			
1–3	20(34.5)	29(25.0)	49(28.2)
≥4	31(53.5)	66(56.9)	97(55.7)
Anemia			
Yes	4(6.8)	12(10.4)	16(9.2)
No	54(93.2)	104(89.6)	158(90.8)
Vaginal discharge during the current pregnancy			
Yes	11(18.9)	26(22.4)	37(21.2)
No	47(81.1)	90(77.6)	137(78.8)
Antepartum hemorrhage			
Yes	4(6.8)	11(9.5)	15(8.6)
No	23(39.7)	39(33.7)	62(35.6)
Water like discharge			
Yes	7(12.1)	15(12.9)	22(12.6)
No	24(41.4)	51(43.9)	75(43.2)

### Intrapartum related characteristics

Of the total participants, 16(27.6%) of the mothers with cases and 10(8.6%) of the mothers with controls were undergone augmentation. Nearly one-third 18(31.1%) of the cases and only one-tenth 12(10.4%) of the controls were born from mothers with a history of prolonged labor. Eighteen (31.1%) of the cases and 26(22.4%) of the controls were delivered with meconium-stained amniotic fluid. Twenty four (41.4%) of the cases and 12(10.4%) of the controls had a breech presentation. Similarly, 27(46.5%) of mothers with cases and 22(18.9%) of mothers with controls were not followed by partographs. Furthermore, 6(10.4%) of mothers with cases and 18(15.5%) of mothers with controls had a history of premature rupture of membrane.

### Fetal related characteristics

Out of the total newborns, 93(53.5%) were male. Among these 33(56.9%) of them were cases and 60(51.7%) controls. About 5(8.6%) of the cases and 14(12.1%) of the controls were preterm. Twenty-two (37.9%) of the cases and 12(10.3%) of the controls were low birth weight. The majority (94.8%) of the cases and (89.7%) of the controls were singleton. Four (6.9%) of the cases and 5(4.3%) of the controls had fetal distress.

### Determinants of birth asphyxia

Bi-variable logistic regression analysis showed that 12 variables were associated with birth asphyxia. After adjustments for possible effects of confounding variable, prolonged labor, caesarean section (CS), vaginal assisted delivery, breech presentation, fail to use partograph, and low birth weight was identified to be determinants of birth asphyxia. The odds of birth asphyxia among newborns born from mothers who were not followed by partograph (AOR = 3.36, 95% CI: 1.99, 13.21) during labor was 3.36 times higher compared to those who were followed by partograph. Newborns born with low birth weight (AOR = 3.74, 95% CI: 1.48, 9.37) were 3.74 times more likely to develop birth asphyxia compared to normal birth weight. Similarly, newborns delivered by CS (AOR = 3.68, 95% CI; 1.31, 10.28) and vaginal assisted (AOR = 5.69, 95% CI; 2.17, 14.91) were 3.68 and 5.69 times more likely to develop birth asphyxia than those who delivered via spontaneously vaginal delivery respectively. The newborns born from mothers with prolonged labor (AOR = 4.15, 95% CI: 1.56, 11.06) and breech presentation (AOR = 5.13, 95% CI: 1.99, 13.21) had also 4.15 and 5.13 times more likelihood of birth asphyxia than those who had normal labor and cephalic presentation respectively (Table 3).

**Table 3:** Bi-variable and multivariable logistic regression analysis of factors associated with birth asphyxia, West Shoa Zone, Central Ethiopia, 2020.

Variables	Cases (n = 58)	Controls (n = 116)	COR (95%CI)	AOR (95%CI)	P-value
Occupation					
Housewife	25(43.2)	51(43.9)	1	1	
Merchant	19(32.8)	34(29.3)	1.14(0.54, 2.38)	1.06(0.38, 2.98)	0.902
Self-employee	3(5.1)	13(11.2)	0.47(0.12, 1.80)	0.47(0.09, 2.47)	0.375
Government employee	3(5.1)	14(12.1)	0.43(0.11, 1.66)	0.26(0.05, 1.23)	0.101
Student	8(13.8)	4(3.5)	4.08(1.12, 14.85)	1.66(0.35, 7.89)	0.523
Type of labor					
Spontaneous	34(58.6)	98(84.5)	1	1	
Augmented	16(27.6)	10(18.6)	4.61(1.91, 11.13)	2.18(0.71, 6.67)	0.170
Induced	8(13.8)	8(6.8)	2.88(1.00, 8.27)	0.81(0.18, 3.54)	0.781
Duration of labor					
Normal	40(68.9)	104(89.6)	1	1	
Prolonged	18(31.1)	12(10.4)	3.91(1.72, 8.82)	**4.15(1.56, 11.06)**	0.004
Complication of labor					
No	46(79.3)	103(88.8)	1	1	
Yes	12(20.7)	13(11.2)	2.06(0.87, 4.87)	0.54(0.17, 1.68)	0.294
Cord prolapsed					
No	53(91.4)	113(97.4)	1	1	
Yes	5(8.6)	3(2.6)	2.79(0.60, 12.90)	1.68(0.14, 19.45)	0.670
Nuchal cord					
No	55(94.9)	115(99.2)	1	1	
Yes	3(5.1)	1(0.8)	6.27(0.64, 61.68)	3.76(0.23, 59.49)	0.341
Labor attendant					
Midwife	23(39.6)	81(69.8)	1	1	
Integrated surgical officer	24(41.5)	24(20.7)	3.52(1.69, 7.31)	1.29(0.39, 4.21)	0.671
General practitioner	4(6.8)	3(2.6)	4.69(0.98, 22.50)	3.87(0.46, 32.02)	0.209
Obstetrician	7(12.1)	8(6.9)	3.08(1.01, 9.39)	0.69(0.14, 19.45)	0.680
Meconium stained amniotic fluid					
No	40(68.9)	90(77.6)	1	1	
Yes	18(31.1)	26(22.4)	1.56(0.76, 3.14)	0.98(0.34, 2.79)	0.970
Fetal presentation					
Cephalic	34(58.6)	104(89.6)	1	1	
Breech	24(41.4)	12(10.4)	6.11(2.76, 12.53)	**5.13(1.99, 13.21)**	0.001
Partograph use					
Yes	31(53.5)	94(81.1)	1	1	
No	27(46.5)	22(18.9)	3.72(1.86, 7.45)	**3.36(1.44, 7.79)**	0.005
Mode of delivery					
Spontaneous vaginal delivery	26(44.8)	93(80.2)	1	1	
Vaginal assisted delivery	15(25.9)	10(8.6)	5.36(2.15, 13.34)	**5.69(2.17, 14.91)**	0.001
Caesarean section	17(29.3)	13(11.2)	4.67(1.01, 10.86)	**3.68(1.31, 10.28)**	0.013
Birth weight					
Normal	36(62.1)	104(89.7)	1	1	
Low	22(37.9)	12(10.3)	5.29(2.38, 11.77)	**3.74(1.48, 9.37)**	0.005

## Discussion

Asphyxia ‘insufficient oxygen supply’ can lead to severe hypoxic-ischemic organ damage in newborns followed by a fatal outcome or severe life-long pathologies. Hence, the quality of obstetric care during pregnancy, intrapartum and postpartum periods are crucial to reduce the overall newborns’ morbidity and mortality. Prolonged labor, breech presentation, mode of delivery: cesarean section and assisted vaginal birth, not following labor with partograph, and low birth weight were found to be determinants of birth asphyxia. This result provides important information with regard to determinants of birth asphyxia and complements the limited data in the study setting, which is valuable to develop contextual interventions.

In the present study, prolonged labor had 4-fold higher odds of birth asphyxia compared to normal labor. This finding is consistent with the studies conducted in Ethiopia, Malawi, and Sweden [37, 38, 43, 44]. If labor fails to progress, it makes newborns involved in labor for a long time which increases the chance of birth trauma, and any attempt to speed up delivery using oxytocin, forceps delivery or vacuum extraction [45]. This implies the importance of early diagnose and management of the first and second stage of labor disorders to minimize the outcomes of birth asphyxia [13].

Newborns with the breech presentations had 5-fold higher odds of birth asphyxia compared to cephalic presentations. This finding is comparable with the studies done in Bangladesh, Pakistan, and Iraq [46–48]. This is because breech presentation increases the chance of umbilical cord compression and prolapse which may lead to birth asphyxia [46]. This finding necessitates obstetric care providers to prepare themselves for the management of birth asphyxia when the fetal presentation is non-cephalic.

Newborns delivered by cesarean section and assisted vaginal birth had 4 and 6-folds higher odds of birth asphyxia compared to those who delivered through spontaneous vaginal delivery respectively. This finding is similar to the studies conducted in Pakistan, and Gondar, Dessie, and Asella [17, 37, 40, 49]. This is because either most mothers came late with the complications of labor or the decision for CS delayed that would increase the burden of birth asphyxia [50]. The other possible explanation is fetal chest might be pressed when the newborns pass through the birth canal which might evacuate secretion. This in turn decreases the chance of developing birth asphyxia, but this physiological advantage is not seen in CS delivery [51]. And also both vacuum and forceps extraction exert pressure on the newborn’s brain and it might cause the brain to bleed on the cranium contributes to intracranial hemorrhage and birth asphyxia [52]. This finding indicates the cautious analysis and decision of interventions during intrapartum care to reduce unnecessary indications of assisted vaginal birth and cesarean section to minimize the magnitude of birth asphyxia [13].

Newborns delivered from mothers who were not followed by partograph had 3-fold higher odds of birth asphyxia compared to those who were followed by partograph. This finding is in line with the study done in India [53]. The possible explanation might be, following labor with the partograph helps providers to monitor the progress of labor, maternal, and fetal condition which alarms labor attendants to take necessary steps if complications occur. Hence, if labor is not followed by partographs it is more likely that newborns develop birth asphyxia [54]. This finding implies the benefit of partograph to reduce newborns morbidity and mortality in the resource-limited region.

Moreover, low birth weight had 4-fold higher odds of birth asphyxia compared to normal birth weight. This finding is consistent with the studies conducted in Gondar, Tigray, and Bangladesh [37, 47, 55]. This might be due to the fact that low birth weight can be caused by maternal medical conditions such as hypertension and diabetes mellitus that would increase the burden of birth asphyxia [56]. This specifies that health care providers should give more attention to newborns with low birth weight and give necessary care before complications occur.

### Limitation of the study

Firstly, since the study depends on APGAR score to classify both cases and controls there might be a chance of misclassification. Secondly, although the authors carefully identified the study population and the choice of the right comparison in hopes of creating a more representative sample, selection and information bias may still happen and made the results non-generalizable. This study might fill a variety of gaps in information regarding the determinants of birth asphyxia in the study population and helps policymakers to consider contextual interventions.

## Conclusions

In this study; prolonged labor, failure to use partograph, breech presentation; CS and vaginal assisted delivery, and low birth weight were the determinants of birth asphyxia. Thus, health care providers should follow the progress of labor with partograph to early identify prolonged labor, fetal presentation and determine the mode of delivery that would minimize the burden of birth asphyxia.

## Supporting information

S1 File (DOCX)

S2 File (DOCX)

S1 Dataset (SAV)

## Abbreviations

APGAR: Appearance, Pulse rate, Grimaces, Activity, and Respiration
CS: Caesarean Section
MUAC: Mid upper arm circumference
