# Supplementary material for: Determinants of birth asphyxia among newborns delivered in public hospitals of West Shoa Zone, Central Ethiopia: A case-control study
Source: PLoS One. 2021 Mar 16;16(3):e0248504. doi: 10.1371/journal.pone.0248504 (PMC7963050; doi:10.1371/journal.pone.0248504)
Supplement: S1 File — (DOCX) [file pone.0248504.s001.docx]

**S1 File.**

**Ajaja 1ffaa: Gaaffillee waa’ee haala jiruufi jireenya/ hawwaasummaa/haadholiiq orannoo irratti hirmaataniin walqabate Haadholii qorannoo irratti hirmaataniif gaaffi gochuun deebii filannoo isaaniitti mari**

| ID | Gaaffilee | Deebiifilannoo | Gara ittiaanutti darbi |
| --- | --- | --- | --- |
| 101 | Umuriinkeessanmeeqa? | ________(waggaadhaan) |  |
| 102 | Haalagaa’elaa? | 1. Kanhinheerumne 2. Kanheerumteabbawarraawaliinjirtu 3. Kanhiikte/ addabaate 4. Kanabbaanwarraajalaadu’e |  |
| 103 | Sabummaankeessanmaali? | 1. Oromoo 2. Amharaa 3. Guraagee 4. Tigiree 5. Kan biro (addabaasi) |  |
| 104 | Amantaankeessanmaali? | 1. Ortodoksii 2. Proteestaantii 3. Musliima 4. Waaqeffataa 5. Kaatolikii 6. Kan biro (addabaasi) |  |
| 105 | Iddoonjireenyaakeessaneessa? | 1. Magaalaa 2. Baadiyyaa |  |
| 106 | Sadarkaanbarnootaakeessanhangami? | 1. Barumsaammayyaakanhinqabne 2. Sadarkaajalqabaa / 1-8/ 3. Sadarkaa 2 ^ffa^ (9-12) 4. Kooleejii fi isaaol |  |
| 107 | Hojiinkeessanammaamaali? | 1. Haadhawarraa 2. Daldala 3. Hojiidhunfaa 4. Hojiimootummaa 5. Barattuu 6. Hojjetaaguyyaa 7. Dhaabbatamootummaanalaa (NGO) 8. Kan biro (addabaasii) |  |
| **Ajaja2ffaa:Gaaffileewaa’eeyerooulfaa fi da’umsaduraanwalqabatan** | | | |
| ID | Gaaffilee | Deebiifilannoo | Gara ittiaanutti darbi |
| 201 | Ijoolleemeeqadeesse? | _______________ |  |
| 201 | Rakkoonda’umsaisaduraairrattisimudatejiraadaa’imaankanwalqabate? | 1. Eeyyee 2. Lakkii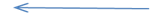 | Deebiinlakkiiyoota’eGarG204tti darbi |
| 203 | Yoodeebiinkeessaneeyyeeta’eG 202irratti, rakkoonmudatemaali? | 1. Ulfiirraaba’u 2. Daa’imniakkadhalateendu’u 3. Daa’imniergatureedu’u 4. Daa’imniulfaatinanxiqqaadhalachuu 5. Daa’imniyeroonutuuhinga’indhalachuu(preterm) 6. Daa’imniyeroonirradarbedhalachuu(postterm) 7. Kanbiroo(addabaasi) |  |
| 204 | Ulfakeessanammaakanaafhordoffiigootaniittuu? | 1. Eeyyee 2. Lakkii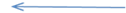 | Deebiinlakkiiyoota’eGara G 206tti darbi |
| 205 | Yoodeebiinkeessaneeyyeeta’eG204irrattiyeroomeeqaafdeddeebitaniihordoffiiraawwattan? | _____________(lakkoofsaankaa’i) |  |
| 206 | Dhukkubniyeroodheraafisindhukkubaaturejiraa?(chronic illness) | 1. Eeyyee 2. Lakkii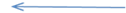 | Deebiinlakkiiyoota’eGara G 208tidarbi |
| 207 | Deebiinkeessan G 206tiifeeyyeeyoota’emaalture? | 1. Dhiibbaadhiigaa 2. Dhibeesukkaaraa 3. Dhibeeonnee 4. Dhukkubakalee 5. HIV/AIDS 6. Dhukkubabusaa 7. Kan biro (addabaasi) ) |  |
| 208 | Dhangala’aanyerooulfaakaraaqaamawalhormaatakeessaniindhangalu’ujiraturee? | 1. Eeyyee 2. Lakkii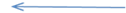 | Deebiinlakkiiyoota’eGaraAjaja III tidarbi |
