# Supplementary material for: Determinants of birth asphyxia among newborns delivered in public hospitals of West Shoa Zone, Central Ethiopia: A case-control study
Source: PLoS One. 2021 Mar 16;16(3):e0248504. doi: 10.1371/journal.pone.0248504 (PMC7963050; doi:10.1371/journal.pone.0248504)
Supplement: S2 File — (DOCX) [file pone.0248504.s002.docx]

## S2 File.

## English version questionnaire

**Identification related information**

Data collector’s Signature: ______________________Date___________________________

Supervisor’s Signature: ________________________Date____________________________

Questionnaire Code No: ____________________ Hospital Name: _______________________

**Part I: Now, I am going to ask you some questions about your socio-demographic status**

| ID | Questions | Code of variables |
| --- | --- | --- |
| 101 | How old are you? | _____________(in complete years) |
| 102 | What is your marital status? | 1. Single 2. Married/ live together 3. Divorced/ separated 4. Widowed |
| 103 | What is your ethnicity? | 1. Oromo 2. Amhara 3. Gurage 4. Tigre 5. Other( specify) |
| 104 | What is your religion? | 1. Orthodox 2. Protestant 3. Muslim 4. Wakefata 5. Catholic 6. Other(specify) |
| 105 | What is your residence? | 1. Urban 2. Rural |
| 106 | What is you educational status? | 1. No formal education 2. Primary school / 1-8/ 3. Secondary (9-12) 4. college and above |
| 107 | What is your current occupation? | 1. House wife 2. Merchant 3. Private employee 4. Government employee 5. Student 6. Daily laborer 7. NGO 8. Others( specify) |

**Part II: Now, I would like to ask you some questions regarding your obstetrics and medical conditions (maternal and antepartum factors).**

| ID | Questions and guides to data collectors | Code of variables | Skip |
| --- | --- | --- | --- |
| 201 | How many times you have been delivered (parity)? | _______________ |  |
| 202 | Did you have previous history of adverse pregnancy outcome? | 1. Yes 2. No | If no Skip Q204 |
| 203 | If yes to Q 202 what type? | 1. Abortion 2. Still birth 3. Child death 4. Low birth weight neonate 5. Preterm neonate 6. Post term neonate 7. Others(specify) |  |
| 204 | Did you visit ANC in current pregnancy? | 1. Yes 2. No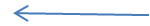 | If no Skip to  Q 206 |
| 205 | If yes to Q 204how many times you visited? | ____________( put in number) |  |
| 206 | Do you have any chronic medical illness during current pregnancy? | 1. Yes 2. No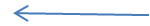 | If no skip to Q 208 |
| 207 | If yes to Q 206which type? (multiple response is possible probe for further responses, circle all that applies) | 1. Hypertension 2. Diabetes mellitus 3. Heart disease 4. Kidney disease 5. HIV/AIDS 6. Malaria 7. Others(specify) |  |
| 208 | Is there any discharge through vagina during current pregnancy? | 1. Yes 2. No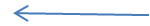 | If no skip to part III |
| 209 | If yes for Q208 which one ?(multiple response is possible probe for further responses, circle all that applies) | 1. APH (bleeding in pregnancy) 2. Watery vaginal discharge 3. Others (specify) |  |

**Part III: Questions to be filled from medical records or by Measurement**

| Code | Check list and guides for data collectors | Possible response | Skip |
| --- | --- | --- | --- |
| 301 | How did your labor started? | 1. Spontaneous 2. Augmented 3. induced |  |
| 302 | How long did the labor take? | _________(in hour) |  |
| 303 | I there any Complication during labor? | 1. Yes 2. No | If no skip to Q 305 |
| 304 | If yes to Q 303 what are they? (Multiple responses are possibleprobe for further responses, circle all that applies*)***).** | 1. Preeclampsia 2. Eclampsia 3. Uterine rupture 4. Nuchal cord 5. Cord prolapse 6. Placental abruption 7. Excessive bleeding 8. Maternal fever 9. Others(specify) |  |
| 305 | What is APGAR score at 1^st^ minute? | _____________ |  |
| 306 | What is APGAR score at 5^th^ minute? | _____________ |  |
| 307 | Did amniotic fluid is meconium stained during delivery? | 1. Yes 2. No |  |
| 308 | Did membrane rupture is premature rupture? (It is considered when rupture of the membrane occurs one hour before the onset of labor? | 1. Yes 2. No |  |
| 309 | Did rupture membrane is prolonged rupture? (It is considered when rupture of membranes was 18 hours before the birth of baby) | 1. Yes 2. No |  |
| 310 | Is there Iron deficiency anemia? | 1. Yes 2. No |  |
| 311 | What is fetal presentation of new born? | 1. Cephalic 2. Breech |  |
| 312 | What was mode of delivery? | 1. SVD 2. Vaginal assist 3. C/S |  |
| 313 | Did labor is followed by partograph? | 1. Yes 2. No |  |
| 314 | What is MUAC of mother? | ____________(in centimeter) |  |
| 315 | What is height of mother? | ____________(in centimeter) |  |
| 316 | What is gestational agebased on LMNP or see card for physician Diagnosis or see early ultrasound result? | _________(put in completed weeks) |  |
| 317 | What is fetal heart rate of newborn? | _________(beat/minute) |  |
| 318 | Who did attend the labor? | 1. Midwife 2. Nurse 3. Health officer 4. General practitioner(Gp) 5. IESO 6. Obstetrician |  |
| 319 | What is birth weight of newborn? | _________( in kilogram) |  |
| 320 | Number of newborn delivered? | ___________ |  |
| 321 | What is sex of newborn? | 1. Male 2. Female |  |
